# Derivation and Preclinical Characterization of CYT-303, a Novel NKp46-NK Cell Engager Targeting GPC3

**DOI:** 10.3390/cells12070996

**Published:** 2023-03-24

**Authors:** Antonio Arulanandam, Liang Lin, Hao-Ming Chang, Martine Cerutti, Sylvie Choblet, Peng Gao, Armin Rath, Armand Bensussan, Jean Kadouche, Daniel Teper, Ofer Mandelboim, Wei Li

**Affiliations:** 1 Cytovia Therapeutics, Inc., Natick, MA 01760, USA; 2 Baculovirus and Therapy, UAR3426 Biocampus, Centre National De La Recherche Scientifique (CNRS), 34293 Montpellier CEDEX 5, France; 3 CLCC de Reims, U976 and Institut Godinot, The Institut National de la Santé et de la Recherche Médicale (Inserm), 1 Rue Du General Koenig, 51726 Reims CEDEX, France; 4 The Lautenberg Center for Immunology and Cancer Research, Institute for Medical Research Israel-Canada (IMRIC), Faculty of Medicine, Hebrew University of Jerusalem, P.O. Box 12272, 91120 Jerusalem, Israel

**Keywords:** natural killer cells, NK engager, bispecific antibody, NKp46, GPC3, hepatocellular carcinoma

## Abstract

Glypican-3 (GPC3) is an oncofetal antigen that is highly expressed in multiple solid tumors, including hepatocellular carcinoma, and is barely expressed in adult normal tissues except the placenta. NKp46 activation receptor is expressed in all-natural killer (NK) cells, including tumor-infiltrating NK cells. FLEX-NK^TM^ is a platform for the production of tetravalent multifunctional antibody NK cell engagers (NKE). CYT-303 was designed using the FLEX-NK scaffold, incorporating a novel humanized NKp46 binder that does not induce NKp46 internalization and a humanized GPC3 binder that targets the membrane-proximal lobe to mediate NK cell-redirected killing of HCC tumors. CYT-303 shows sub-nanomolar binding affinities to both GPC3 and NKp46. CYT-303 was highly potent and effective in mediating NK cell-redirected cytotoxicity against multiple HCC tumor cell lines and tumor spheroids. More interestingly, it can reverse the dysfunction induced in NK cells following repeated rounds of serial killing of tumors. It also mediated antibody-dependent cellular phagocytosis (ADCP) and complement-dependent cytotoxicity against GPC3-expressing HCC tumors. In vivo, CYT-303 showed no toxicity or cytokine release in cynomolgus monkeys up to the highest dose (60 mg/kg), administered weekly by intravenous infusion for 28 days. These results demonstrate the potential of CYT-303 to be a safe and effective therapy against HCC.

## 1. Introduction

Natural killer (NK) cells are the prototype innate lymphoid cells endowed with a potent cytolytic function that provides host defense against microbial infection and tumors. They express a variety of activating and inhibitory receptors that regulate their functions. NK cells can recognize and then spontaneously kill “stressed” cells, such as tumor cells, without prior sensitization. Blood NK cell counts positively correlate with a lower risk for cancer development, whereas higher tumor tissue NK cell infiltration correlates with improved treatment outcomes [1]. Unlike T cell-based therapies, NK cells elicit little if any cytokine release syndrome (CRS), immune effector cell-associated neurotoxicity syndrome (ICANS), or graft versus host disease (GvHD), but produce potent anti-tumor clinical responses in non-Hodgkin lymphoma patients [2]. NK cells can be activated by their activating receptors and exert antibody-dependent cell cytotoxicity (ADCC) by recognizing antibody-coated cells through the low-affinity receptor for the Fc portion of IgG1 antibodies (FcγRIIIa or CD16). Upon activation, NK cells release lytic granules containing perforin and granzyme B within the immunological synapse to kill target cells. They also secrete soluble factors such as tumor necrosis factor α (TNF-α), TNF-related apoptosis-inducing ligand (TRAIL), and Fas ligand (FasL) to trigger apoptosis in target cells. Finally, they secrete interferon γ (IFN-γ), growth factors (GM-CSF), immunoregulatory cytokines (IL-15, IL-10, and IL-13), and chemokines. These cytokines modulate both innate and adaptive immune responses, such as dendritic cell (DC) maturation and CD4^+^ to Th1 T cell differentiation, respectively [1].

NKp46 (CD335), an activation receptor, is one of the earliest natural cytotoxicity-triggering receptors identified (NCR1) and is a highly specific marker of NK cells [3,4]. It is expressed in all NK cells and some ILCs, including tumor-infiltrating NK cells, while many of the other NK activating receptors are downregulated in the suppressive tumor microenvironment [5]. NKp46 ligation is sufficient to induce Ca2+ increases, lymphokine production, and cytolytic activity in NK cells [4,6]. As with CD16, it triggers potent signaling pathways via ITAM, which contains subunits CD3 zeta (CD3ζ) and Fc receptor gamma (FcRγ), to activate Zap70, Syk, and PI3K kinase to mediate NK cytotoxicity and cytokine production [7]. Therefore, utilizing NKP46 to engage NK cells may be more effective for solid tumor indications.

**Figure 1:**
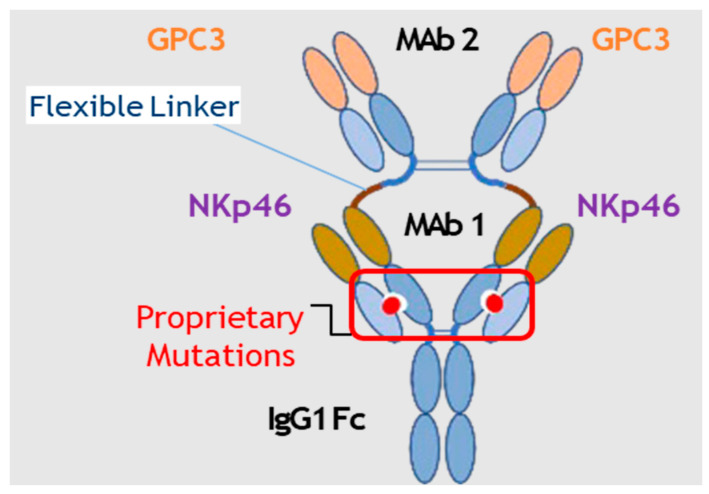
Diagram of the structure of CYT-303.

Our FLEX-NK^TM^ platform utilizes a bispecific, multifunctional antibody scaffold previously described by Golay [8]. This scaffold leverages a tetravalent format to improve the affinity, avidity, and specificity of the binding to targets. It also uses flexible peptide linkers to fuse the VH-CH1 domains of a first mab (Figure 1, mAb2) to the H chain of a second mab (Figure 1, mAb1) to allow simultaneous binding of two targets on different cells. It contains a fully functional Fc region, which confers a characteristic IgG1 half-life on the antibody and facilitates additional effector function via CD16 expressed on NK cells and macrophages, as well as complement activation. In addition, mutations were introduced in the CH1 and CL1 interfaces of mAb1 to ensure the correct pairing of the light chains to their corresponding mAb1 and mAb2 VH-CH domains. 

GPC3 is a glycophosphatidylinositol (GPI)-anchored cell surface heparan sulfate proteoglycan that is expressed during early development. Its expression can be detected in human embryos, fetuses, and placental tissues but not in normal adult tissue or livers with benign diseases [9]. GPC3 is highly expressed in 70–100% of hepatocellular carcinomas (HCC). It interacts with Wnt to facilitate Wnt/Frizzled binding and signaling for HCC growth. GPC3 expression on HCC tumor cells is correlated with a poor prognosis in HCC [10,11,12]. Therefore, most HCC patients who have received prior systemic therapy are likely to express GPC3 on their tumor cells. Despite the availability of multiple therapeutic options, no curative care exists for patients with advanced or metastatic HCC. Average progression-free survival is less than 7.5 months, and the 5-year survival rate is less than 9% [13,14]. Current treatments include immunotherapies such as checkpoint blockade monoclonal antibodies directed against programmed cell death protein 1 (PD-1), programmed cell death ligand 1 (PD-L1), or cytotoxic T-lymphocyte associated protein 4 (CTLA-4), antiangiogenic monoclonal antibodies, as well as multi-kinase inhibitors such as bevacizumab, sorafenib, lenvatinib, and cabozatinib, but effective systemic treatment options for HCC are still limited. NK cells have been shown to infiltrate into HCC tumors, but their activities are downregulated due to various suppressive signals in the tumor microenvironment [15,16]. Here we tested whether CYT-303, a bispecific multifunctional antibody that utilizes NKp46 to engage NK cells and GPC3 to target HCC, can induce NK cell cytotoxicity, cytokine production, ADCP, and CDC to kill HCC tumors and have the potential to be developed as a clinically relevant therapeutic against HCC.

## 2. Materials and Methods

### 2.1. Cell Lines

Hep3B (human hepatocarcinoma), HepG2 (human hepatoblastoma), Huh-7 (human hepatocarcinoma), BW (murine T-lymphoblast), BJAB (EBV-negative human Burkitt-like lymphoma), MCF7 (human breast cancer), and C1R (human B-cell lymphoblastoid) cell lines used in this study were purchased from ATCC (Manassas, VA, USA). BW NKp46 cells were generated by stably transfecting NKp46 into BW cells [17], and Hep3B-GFP cells were generated by stably transfecting GFP into Hep3B cells. 

### 2.2. Antibodies

PE mouse anti-human IgG and APC-H7 mouse anti-human CD3 were purchased from BD (Franklin Lakes, NJ, USA). Goat anti-human IgG1 PE was purchased from Thermo Fisher Scientific (Waltham, MA, USA). PE/Cyanine7 anti-human IFN-γ antibody, PE anti-human TNF-α antibody, APC anti-human CD107a (LAMP-1) antibody, APC anti-human CD56 (NCAM) antibody, PE anti-human CD16 antibody, PE anti-human CD335 (NKp46) antibody (9E2), Brilliant Violet 421™ anti-human CD45 antibody, PE/Cyanine7 anti-human CD14 antibody, PE anti-human CD19 antibody, and Zombie NIR™ Fixable Viability Kit were purchased from Biolegend (San Diego, CA, USA). 

### 2.3. Generation of Anti-NKp46 and GPC3 Monospecific Antibodies and CYT-303 Bispecific Antibodies

The anti-NKp46 mAb (09) was generated by injecting NKp46-deficient Ncr1^gfp/gfp^ mice [3] with NKp46-Ig fusion proteins. Humanization of the anti-NKp46 mAb 09 was conducted using the automated humanization software from Macromoltek (Austin, TX, USA). All antibodies are of the IgG1 isotype. Three plasmids provided by ProteoNic were used to express CYT-303, one to express the heavy chain and the other two to express the two light chains. The two light chains were co-expressed from the same vector, and two vector variants were used for the light chain expression with different gene orders to ensure the equal expression level of the two light chains. 

### 2.4. CYT-303 Binding to NKp46 and GPC3 Recombinant Proteins

Human NKp46 or GPC3 were immobilized on Octet chips, and different concentrations of CYT-303 flowed through the chips, and binding was assessed by Octet Bio-Layer Interferometry (Goettingen, Germany). Kd, Kon, and Koff were calculated by Octet binding analysis software.

### 2.5. PBNK Isolation

PBNKs were isolated from healthy blood donors by negative selection using the RosetteSep™ Human NK Cell Enrichment Cocktail (Stemcell Technologies, Cambridge, MA, USA). Purified PBNKs were expanded in the presence of 500 U/mL r-IL-2. 

### 2.6. Anti-NKp46 mAb and hNKp46-Ig Binding Studies on NKp46 Expressing Cells and Human Tumor Cell Lines, Respectively

Anti-NKp46 mAbs 09 and 9E2 binding to PBNK cells or BW NKp46 transfectants or parental BW cells incubated at 4 °C or 37 °C was detected using a FITC labeled anti-mouse IgG secondary antibody by flow cytometry. hNKp46-Ig was constructed by fusing the NKp46 extracellular domain to human IgG Fc, as previously described [18]. hNKp46-Ig binding to human tumor cell lines was detected either in the presence or absence of anti-NKp46 mAbs 09 or 9E2 by flow cytometry using a PE-labeled anti-human IgG antibody.

### 2.7. CYT-303 Binding PBNK and Tumor Cells

CYT-303 was incubated with PBNK or Hep3B tumor cells for 1 h at 4 °C, and the binding was detected using goat anti-human IgG1 PE antibody by flow cytometry.

### 2.8. PBNK Cytolysis of HCC Tumor Cells

PBNKs were incubated with Hep3B tumor cells at a fixed E/T ratio of 1 for 5 h at 37 °C with varying concentrations of CYT-303 or monospecific anti-NKp46 or GPC3 antibodies and human IgG isotype controls, and target tumor cell lysis was evaluated by flow cytometry using a cell viability dye.

### 2.9. PBNK Degranulation in the Presence of HCC Tumor Cells

PBNKs were incubated with Hep3B tumor cells at a fixed E/T ratio of 1 for 5 h at 37 °C with varying concentrations of CYT-303 or monospecific anti-NKp46 or GPC3 antibodies and human IgG isotype controls, and degranulation of PBNKs was evaluated using an anti-CD107a antibody by flow cytometry.

### 2.10. ADCP and CDC against Hep3B Tumor Cells

Macrophages were differentiated from purified monocytes following culture with M-CSF for 5 days and cultured with Hep3B-GFP tumor cells at an E/T ratio of 1 for 4 h at various concentrations of CYT-303 or human IgG isotype control, and macrophage phagocytosis of tumors was assessed by flow cytometry using a cell viability dye staining of tumors. For complement fixation, the Hep3B-GFP tumor cells were incubated with baby rabbit serum containing complement for 4 h at various concentrations of CYT-303 or human IgG isotype control, and tumor lysis was assessed by flow cytometry using cell viability dye staining of tumor cells.

### 2.11. PBNK Cytolysis of HCC Tumor Spheroids

Hep3B-GFP tumor cells (10^4^ cells/well) were cultured for 2 days in Costar^®^ ultra-low attachment 96-well plates (Thermo Fisher Scientific) to form tumor spheroids and then incubated with PBNKs (10^4^ cells/well) either in the presence of different concentrations of CYT-303 or human IgG for 4 days, and tumor cell cytolysis was monitored over time using the Incucyte^Tm^ Live Analysis System (Goettingen, Germany).

### 2.12. PBNK Serial Killing of HCC Tumor Cells

The serial killing of PBNKs against Hep3B-GFP tumor cells was evaluated with either PBNK cells alone (5 × 10^3^ cells/well) or in combination with different concentrations of CYT-303 or human IgG1 isotype control against and Hep3B-GFP tumor cells (10^4^/well) using a fixed E/T ratio (1:2) at each round of killing. Following each round of tumor cell killing, non-adherent PBNK cells were harvested, counted, and added to fresh Hep3B-GFP tumor cells, and the cytolysis of the tumor cells was monitored by the reduction of GFP-positive tumor cells using the Incucyte^TM^ Live Analysis System (Goettingen, Germany)

### 2.13. In Vitro PBMC Cytokine Release Assay 

Human PBMCs from multiple healthy donors were incubated with different concentrations of CYT-303 or anti-CD3 (Muromonab-CD3) or CD28 mAbs (TGN1412) (Creative Biolabs Inc., New York, NY, USA) as positive controls or hIgG1 isotype controls for 48 h, and supernatants were harvested and tested for the presence of cytokines by multiplex ELISA assay.

### 2.14. In Vitro Fratricide and Immune-Cell Depletion Assessments

NK cell fratricide by CYT-303 was evaluated using purified PBNKs in the presence of CYT-303, Daratumumab, or human IgG by flow cytometry using the live dead cell dye. Human PBMC immune cell subset depletion was evaluated following incubation with CYT-303, Daratumumab, or hIgG1 for 48 h, followed by immune cell subset analysis by flow cytometry.

### 2.15. CYT-303 Pharmacokinetics in Cynomolgus Monkeys

CYT-303 pharmacokinetics in cynomolgus monkeys was incorporated in the single dose range finding tolerability study. Monkeys were administered a single dose of CYT-303 via intravenous infusion, and blood samples were collected over a period of 336 h post-dosing. 

### 2.16. CYT-303 Repeat Dose 4-Week GLP Toxicology Study in Cynomolgus Monkeys 

CYT-303 was administered once weekly for 4 weeks at 6, 20, and 60 mg/kg doses by intravenous infusion, followed by a 6-week recovery period. Clinical signs, cytokine release, cardiovascular safety pharmacology, immunophenotyping, clinical pathology, anatomic pathology, toxicokinetics, and ADA assessments were conducted in the study. 

### 2.17. Pharmacokinetic and Anti-Drug Antibody Assays

NKp46 biotin and GPC3 ruthenium-tagged target capture detection methods were used to develop PK immunoassays and detect CYT-303 exposures in pharmacokinetic and toxicokinetic studies. Streptavidin-coated MSD plates (Mesoscale Discovery, Rockville, MD, USA) were used to capture NKp46 biotin, and an electrochemiluminescence MSD plate reader was used to detect the binding of ruthenium-labeled GPC3. For the antibody assay, anti-CYT-303 antibodies were captured on CYT-303-coated plates and detected using CYT-303 biotin-labeled antibodies in the MSD assay format. A positive control anti-CYT-303 rabbit antibody was used to develop the assay and as a positive control in the assay.

## 3. Results

### 3.1. CYT-303 Design and Biochemical Characterization

Using the FLEX-NK^TM^ platform, CYT-303 is designed according to Golay [8] as a tetravalent bispecific multifunctional antibody comprised of two Fab2 binding domains, each targeting human NKp46 (mAb1) and GPC3 (mAb2), and a fully functional IgG1 Fc domain (Figure 1). Each Fab2 is comprised of variable VH and VL domains and constant CH1 heavy chain and CH1 kappa light chain domains. To achieve correct pairing of mAb1 anti-NKp46 heavy and light chain CH1 domains, structural modeling was used to introduce complementary charged residue mutations to their corresponding CH1 (T192E) and CL1 domains (S114A and N137K), respectively, to enable electrostatic interactions and salt bridge formation. The fusion of the mAb 2 anti-GPC3 VH1-CH1 domains to the H chain of the mAb 1 anti-NKp46 is realized via the hinge region and a flexible peptide linker. The anti-GPC3 binder is hYP7, which is a fully humanized antibody that has been previously described by Phung et al. and Zhang et al. [19,20]. The hYP7 mAb does not inhibit HCC tumor cell proliferation as it binds the C-terminal lobe of GPC3, while the N-terminal lobe of GPC3 is involved in Wnt/Frizzle binding, signaling, and HCC proliferation [21]. The membrane-proximal binding site for CYT-303 is advantageous, as it remains intact following Furin-mediated cleavage and shedding of soluble GPC3 [22].

**Figure 2:**
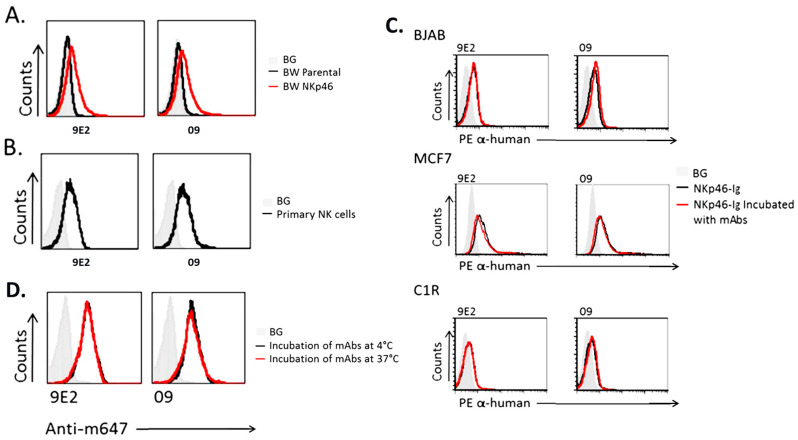
Characterization of the anti-NKp46 mAb 09. (**A**) FACS staining of anti-NKp46 mAbs (9E2 and 09) binding to BW parental cells versus BW transfected cells expressing NKp46 (black and red histograms, respectively). Binding was detected using an anti-mouse IgG AF647 labeled antibody. The filled gray histogram represents staining with secondary antibodies only of the BW parental cells. The background (BG) of BW NKp46 transfectants was similar and is not shown in the figure. The figure shows one representative experiment out of the six performed. (**B**) FACS staining of anti-NKp46 mAbs (9E2 and 09) on primary activated bulk human NK cells (black histogram). The filled gray histogram represents the staining of NK cells with secondary antibodies only. The figure shows one representative experiment out of the six performed. (**C**) Human NKp46-Ig was pre-incubated either alone (black histogram) or with anti-NKp46 mAbs (9E2 and 09, red histograms) at 4 °C, followed by FACS staining of BJAB, MCF7, and C1R cells detected with an anti-human IgG PE-labeled antibody. The filled gray histogram represents the staining of cells with secondary antibodies only. The figure shows one representative experiment out of the two performed. (**D**) Activated bulk NK cell cultures were incubated with the indicated anti-NKp46 mAbs (9E2 and 09) at 4 °C (black histogram) or 37 °C (red histogram) for 8 h, followed by FACS staining with an AF647-labeled anti-mouse secondary antibody. The filled gray histogram represents staining with secondary antibodies only of cells treated at 4 °C. The background of cells treated at 37 °C was similar and is not shown in the figure. The figure shows one representative experiment out of the five performed.

The NKp46 binder used in CYT-303 is based on a novel antibody, 09, against NKp46. This 09 mAb was developed by injecting NKp46-deficient mice with a fusion protein consisting of the extracellular portion of human NKp46 fused to human IgG (NKp46-Ig). It specifically bound BW cells that express NKp46 but not parental BW cells, similar to the binding profile of the commercially available 9E2 NKp46 mAb (Figure 2A). The 09 and 9E2 mAbs showed similar binding profiles against primary PBNK cells (Figure 2B). In order to evaluate if NKp46 binding to its ligand on tumor cells is affected by 09 and 9E2 mAbs, we evaluated the binding of recombinant human NKp46-Ig to tumor cells in the presence or absence of these antibodies. As shown in Figure 2C, the binding of human NKp46-Ig to BJAB, MCF-7, and C1R tumor cells was not affected by the presence or absence of either 09 or 9E2 mAb antibodies, indicating that the NKp46 tumor binding site is distinct from these mAb binding sites. These results suggest that NKp46 activation signals via the tumor ligand interaction could work in concert with the CYT-303 engager signals to redirect PBNK killing of Hep3B tumor cells (Figure 2C). Furthermore, 09 mAb does not downregulate NKp46 expression from the surface of NK cells, which allows for constant engagement of CYT-303 on NK cells to mediate redirected killing of tumors (Figure 2D). The 09 NKp46 antibody was subsequently fully humanized before being incorporated into the FLEX-NK scaffold.

**Figure 3:**
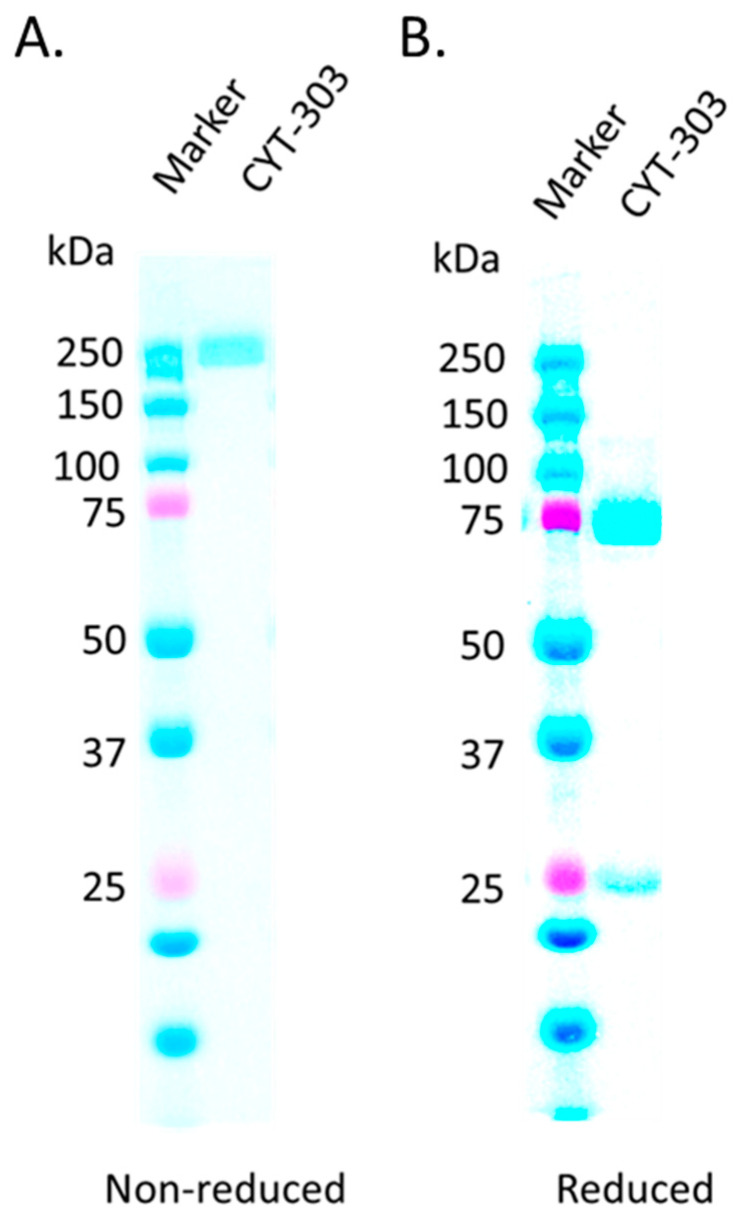
Biochemistry characterization of CYT-303. An amount of 10 μg CYT-303 mixed without (**A**) or with a reducing agent (**B**) in the loading buffer and heated at 70 °C for 10 min. The samples were then loaded onto Bolt™ 4 to 12% Bis-Tris protein gel, electrophoresed until the tracking dye reached the bottom of the gel, and stained with AcquaStain protein gel dye.

Under the non-reducing condition, CYT-303 migrates as a single band (~250 kDa) with a predicted MW of 245 kDa. Under reducing conditions, two protein bands migrating at ~75 kDa and ~25 kDa apparently represent the heavy chain and light chain of CYT-303, with a predicted MW of 74.9 kDa for the heavy chain and 23.4 kDa and 24.3 kDa for the NKp46 and GPC3 light chains, respectively (Figure 3). Although the size of CYT-303 is larger than monoclonal antibodies, our preliminary data indicate that CYT-303 is able to penetrate tumors, and the concentrations in the tumor were above the pharmacologically active concentrations observed in the in vitro NK cell cytotoxicity assays (unpublished data).

### 3.2. Characterization of CYT-303 Binding to Recombinant Target Proteins and Cells

The binding affinity of CYT-303 to human NKp46, GPC3, and CD16a recombinant proteins was evaluated by Octet Bio-Layer Interferometry (BLI). As shown in Table 1, the binding affinity to both GPC3 and NKp46 was in the sub-nanomolar range. The binding affinity to CD16a, as expected, was lower in the sub-micromolar range, with the affinity for the 158 F lower affinity variant being 642 nM and the affinity for the higher affinity 158 V variant being 147 nM. 

**Figure 4:**
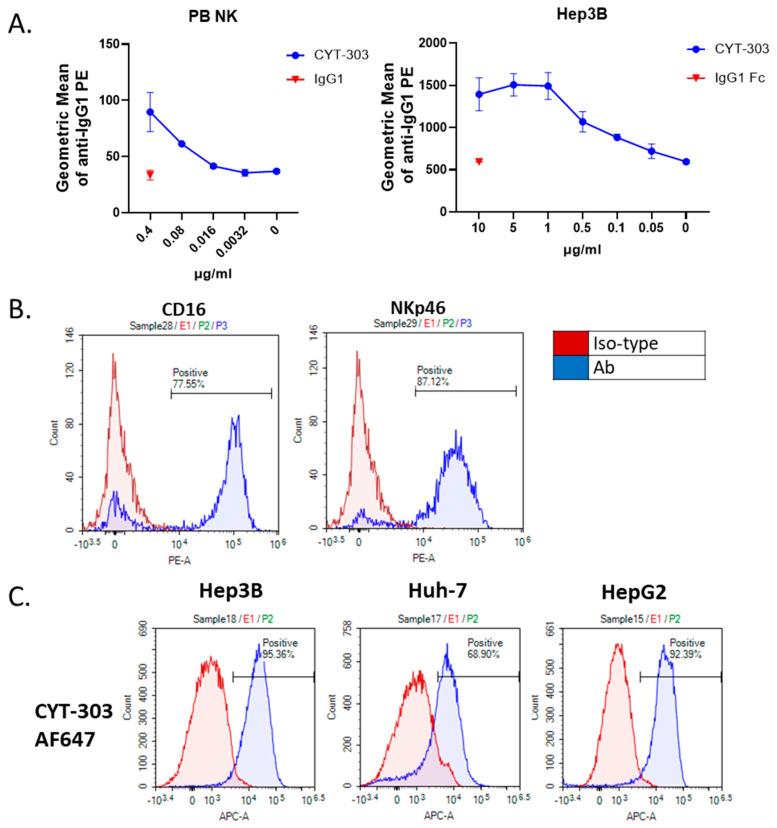
Binding of CYT-303 to PBNK cells and HCC tumor cells. (**A**) Purified PB-NKs or Hep3B tumor cells were incubated with the indicated concentrations of CYT-303 or human IgG1 for 60 min at room temperature (RT), and binding was detected by PE-labeled anti-human IgG by flow cytometry. The graphs are presented as means with standard deviations from two replicates. (**B**) The expression level of CD16 and NKp46 on the purified PBNK cells was assessed using anti-CD16 and anti-NKp46 mAbs. (**C**) GPC3 expression on liver cancer cell lines Hep3B, Huh-7, and HepG2 using AF647 labeled anti-GPC3 mAb by flow cytometry.

The binding of CYT-303 to human PBNK cells that express both CD16a and NKp46 was also evaluated. As shown in Figure 4A, CYT-303 bound PBNK cells in a dose-dependent manner. Additionally, a dose-dependent binding to Hep3B tumor cells was observed. The NKp46 and CD16 expression levels in PBNK are shown in Figure 4B. In addition, the binding of CYT-303 to HCC tumors Hep3B, Huh-7, and HepG2 expressing different levels of GPC3 was evaluated. As shown in Figure 4C, CYT-303 was capable of binding to HCC tumors expressing different levels of GPC3.

### 3.3. CYT-303 Redirected Human PBNK Cytotoxicity against HCC Tumor Cells

**Figure 5:**
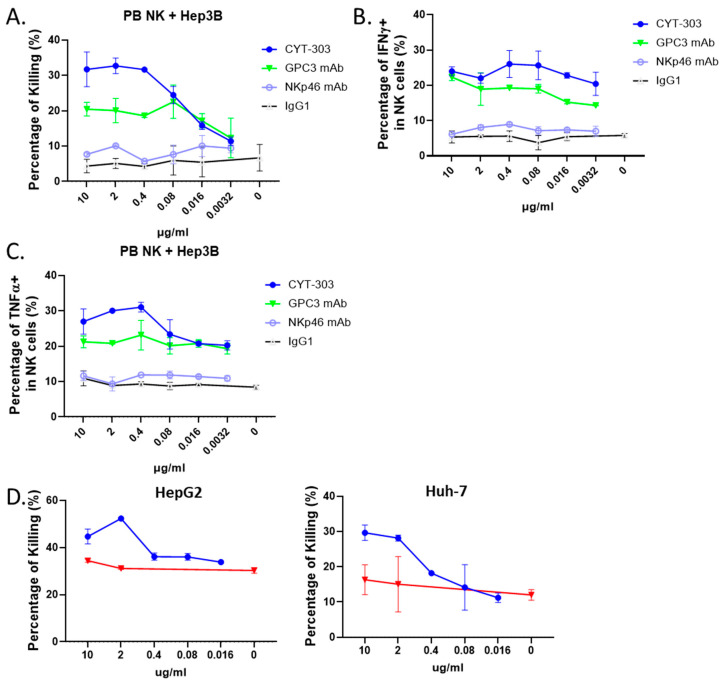
CYT-303 redirected human PBNK cytotoxicity and induced cytokine production. (**A**) CYT-303 redirected the cytolysis of Hep3B at the indicated concentration of CYT-303 at a fixed E/T ratio of 1 for 5 h, as assessed by flow cytometery using a cell viability dye; (**B**,**C**) CYT-303 induced IFNγ and TNFα production of PBNK cells following incubation with Hep3B cells at an E/T ratio of 1 for 5 h were assessed by intracellular staining of PBNKs with anti-IFNγ or TNFα antibodies, respectively. The graph represents data on PBNKs from two donors. (**D**) Cytolysis of HepG2 and Huh-7 tumors. CYT-303 (blue line) or control human IgG (red line) redirected PBNK cell cytolysis of HepG2 (left) and Huh-7 (right) tumors were evaluated at a fixed E/T = 1 for 5 h and assessed by flow cytometry as described above. The graphs are presented as means with standard deviations from two replicates.

CYT-303 redirected PBNK cytolysis, degranulation, and IFNγ and TNFα production in the presence of HCC cells, which were evaluated at an E/T ratio of 1:1 for 5 h. As shown in Figure 5, CYT-303 showed dose-dependent cytolysis, degranulation, and cytokine production against HCC cells, and the maximum effects were observed between 0.4 and 2 mg/mL. The activity of CYT-303 is higher compared to the monospecific GPC3 or NKp46 mAbs, suggesting that co-engagement of NKp46 and GPC3, forming an immunological synapse, is desired for optimal NK cell functions against tumor cells. 

**Figure 6:**
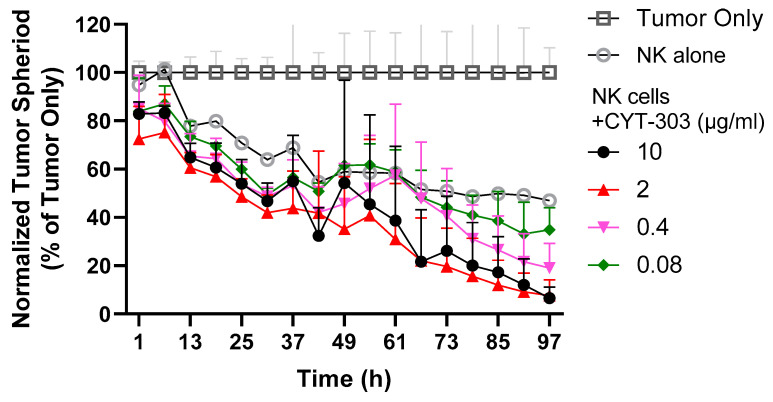
Hep3B-GFP tumor cells were cultured for 2 days in ultra-low attachment U-bottom plates to form tumor spheroids, and then they were incubated with PBNK cells alone or with CYT-303 at the indicated concentrations for 4 days. The remaining tumor spheroids were counted over a period of 4 days. The graph is normalized to the percentage of the tumor only group at each time point and presented as a mean with standard deviation from 3 replicates.

To simulate a solid tumor microenvironment, we tested the activity of CYT-303 in a tumor spheroid assay, and the data showed that the PBNK cell’s killing of Hep3B tumor spheroids was enhanced by CYT-303 in a dose-dependent manner (Figure 6). 

**Figure 7:**
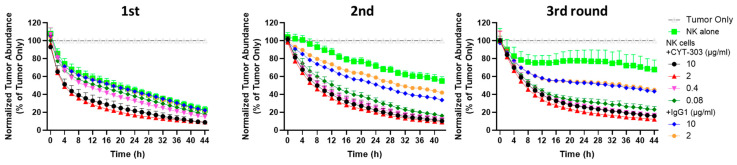
The serial killing of PBNKs against Hep3B-GFP tumors was evaluated with either NK cells alone or in combination with CYT-303 using a fixed E/T ratio (1:2) at each round of killing. Tumor lysis was monitored by the reduction of GFP-positive tumors using the Incucyte Live Analysis System. The graph is normalized to the percentage of the tumor only group at each time point and presented as a mean with a standard deviation from 3 replicates.

We then evaluated the activity of CYT-303 in an NK serial killing assay simulating the ability of NK cells to mediate multiple rounds of killing against tumor cells in the tumor microenvironment. For each round of tumor killing, the same PBNK cells were harvested each time and incubated with freshly added Hep3B tumor cells at the same E/T ratios, and tumor lysis was evaluated in each round of tumor killing for up to 44 h. PBNK cells lost their cytotoxic activity over time in subsequent rounds of serial killing of tumor cells, suggesting that these NK cells become dysfunctional over time. This PBNK dysfunction was completely reversed by CYT-303 in a dose-dependent manner compared to human IgG1 isotype control, suggesting CYT-303-mediated NKp46 and CD16 signaling in NK cells following GPC3 engagement of tumors can mediate potent signaling to reverse NK cell dysfunction (Figure 7).

### 3.4. CYT-303 Mediated ADCP and CDC against HCC Tumor Cells

**Figure 8:**
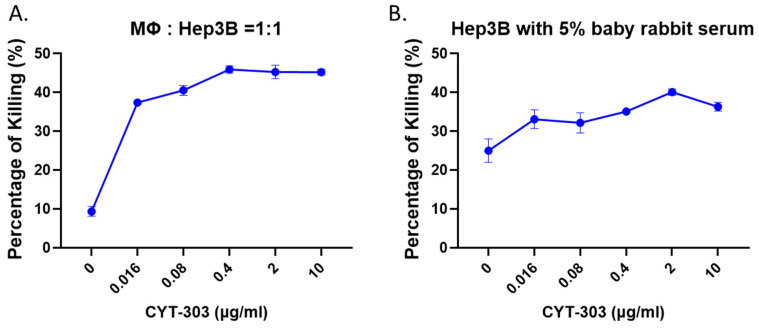
CYT-303 mediates ADCP and CDC in vitro. (**A**) Macrophages were differentiated from purified monocytes following culture with M-CSF for 5 days. CYT-303-induced macrophage phagocytosis of Hep3B-GFP tumor cells was assessed in a 4-h assay using viability dye staining of tumor cells by flow cytometry. The graph is presented as a mean with a standard deviation from three replicates. (**B**) Hep3B tumor cells were incubated with CYT-303 and baby rabbit complement for 4 h, and tumor cell lysis was assessed by flow cytometry using cell viability dye. The graphs are presented as means with standard deviations from four replicates.

The ability of CYT-303 to activate additional macrophage effector cells via CD16 was evaluated in an antibody-dependent cellular phagocytosis assay (ADCP) against Hep3B tumor cells. CYT-303 induced potent macrophage phagocytosis of Hep3B tumor cells in a dose-dependent manner (Figure 8A). Given that the IgG1 isotype of CYT-303 can fix complements, an additional effector function via complement-dependent cytotoxicity (CDC) was evaluated in the presence of rabbit serum containing complement. CYT-303 showed dose-dependent CDC against Hep3B tumor cells, indicating complement fixation and activation of the membrane attack complex (MAC) (Figure 8B). These results show additional effector cell types and mechanisms deployed by CYT-303 to kill HCC tumor cells.

### 3.5. In Vitro Safety Characterization of CYT-303 

**Figure 9:**
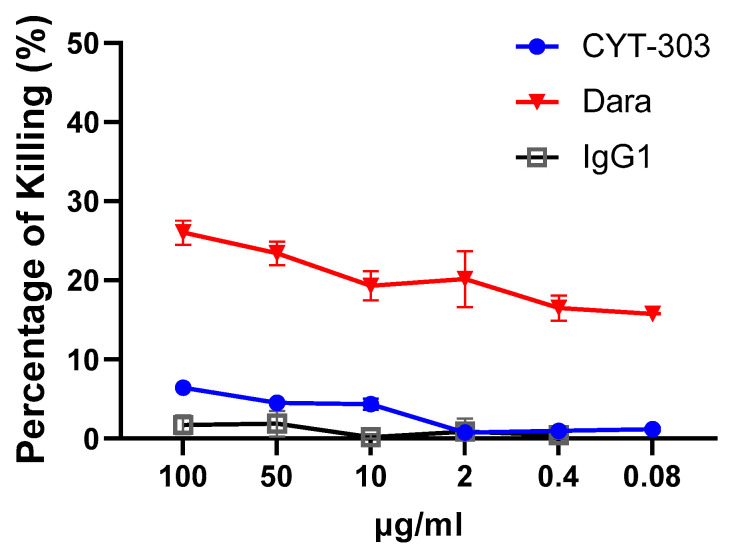
CYT-303 did not induce NK cell fratricide. NK cell fratricide by CYT-303 was evaluated using purified PBNK in the presence of CYT-303, Daratumumab, or human IgG for 24 h. Fratricide was assessed by staining PBNK cells with a live dead cell dye and analyzed by flow cytometry. The graph represents data on PBNKs from two donors.

**Figure 10:**
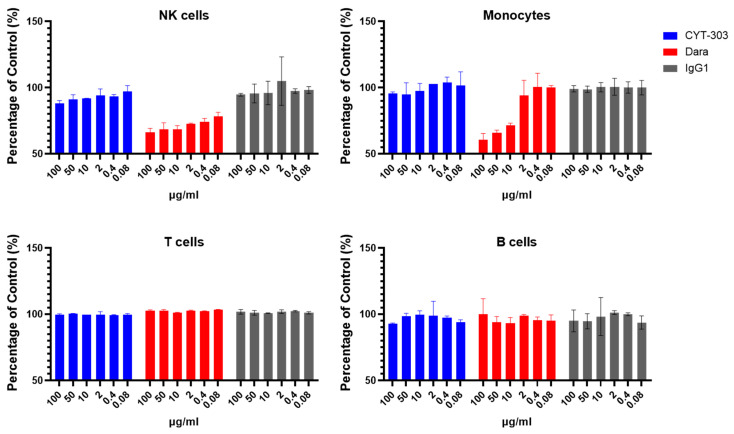
CYT-303 did not induce immune cell depletion. Human PBMCs were incubated with different concentrations of CYT-303, Daratumumab, or human IgG1 for 24 h. Depletion of the immune cell subsets was analyzed by flow cytometry using immune cell subset-specific antibodies and compared to a human IgG control. The graph represents data on PBNKs from two donors.

**Figure 11:**
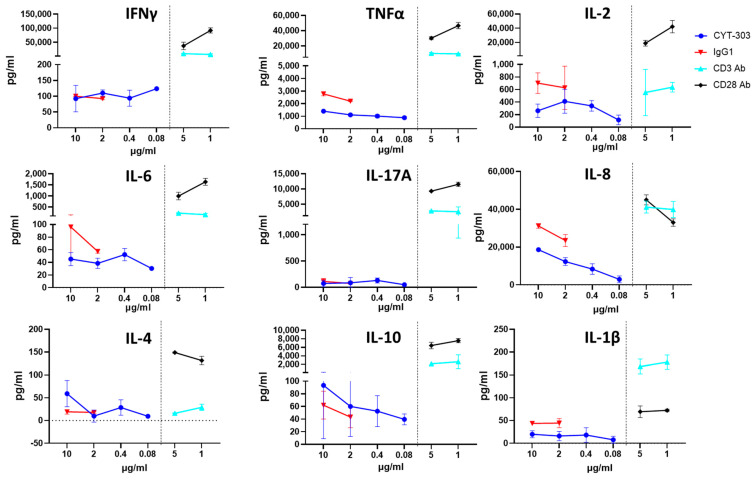
CYT-303 did not induce significant non-specific cytokine release in vitro. Human PBMCs were incubated with indicated concentrations of CYT-303, anti-CD3, or anti-CD28 (TGN1412) mAbs for 48 h. Cytokine release was assessed by a multiplex cytokine immunoassay. The graph represents data on PBNKs from two donors.

As CYT-303 can bind NK cells via either NKp46 or CD16, the possibility of fratricide exists. NK cell fratricide was assessed with different concentrations of CYT-303 or Daratumumab as a positive control or human IgG1 as a negative control. No significant CYT-303-mediated fratricide was observed, while fratricide was readily detected with Daratumumab (Figure 9). In addition, CYT-303-mediated depletion of immune subsets in human PBMC was evaluated. Daratumumab was again used as a positive control. No significant depletion of any immune subsets was observed with CYT-303, while dose-dependent Daratumumab-mediated depletion of NK cells and monocytes was detected (Figure 10). We also evaluated if CYT-303 would induce non-specific cytokine release in vitro with PBMCs from two donors. As shown in Figure 11, while the anti-CD3 and anti-CD28 mAb TGN1412 induced robust cytokine release, no significant cytokine release was observed with CYT-303.

### 3.6. CYT-303 Pharmacokinetics and Toxicology in Cynomolgus Monkeys 

**Figure 12:**
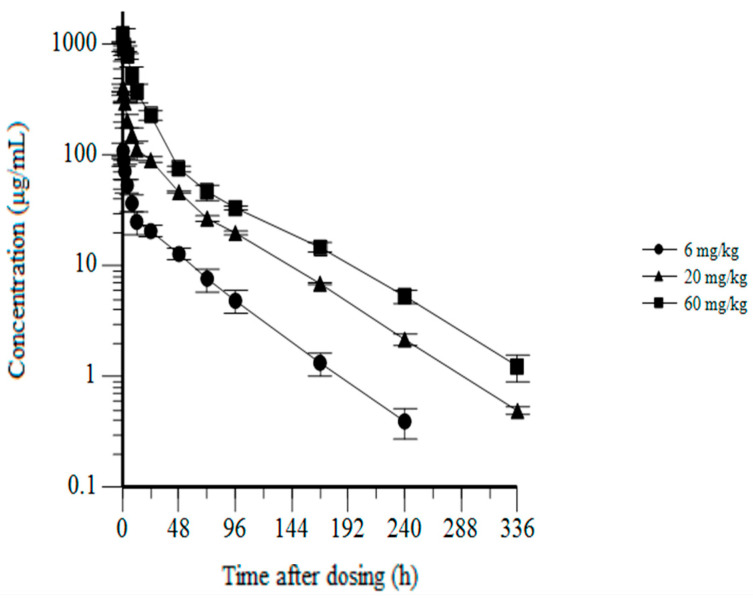
CYT-303 single-dose pharmacokinetics in cynomolgus monkeys. Cynomolgus monkeys were dosed with CYT-303 by intravenous infusion at the indicated dose levels. Blood concentrations were measured up to 336 h post-dosing using a PK immunoassay. Three monkeys per group were evaluated.

To advance CYT-303 into clinical development, the pharmacokinetics and safety of CYT-303 were evaluated in cynomolgus monkeys in a single dose tolerability and pharmacokinetics study followed by a repeat dose. A 28-day GLP toxicity study by weekly intravenous infusions followed by a 42-day recovery phase was conducted to detect potential delayed toxicity and assess the reversibility of any potential toxicities observed. In the single-dose range-finding study, CYT-303 was well tolerated and showed no evidence of any clinical pathology changes, including cytokine release. Pharmacokinetic assessments by non-compartmental analysis showed dose-proportional increases in mean C_max_ and AUC_0–168_ (area under the curve), and mean half-life (t_1/2_) values ranging from 39 to 48 h (Table 2). The pharmacokinetic profiles of CYT-303 are depicted as a log-linear plot and showed exposures lasting for 240–336 h, which could support once-a-week dosing in the first human clinical trials (Figure 12). Moreover, CYT-303 tolerability and pharmacokinetics from the single-dose study allow the selection of CYT-303 doses and weekly dosing frequency for the 28-day GLP toxicity study. 

In the 28-day repeat dose, GLP toxicity studies a total of 32 monkeys (16 males and 16 females) were allocated to 4 dose groups (vehicle, 6, 20, and 60 mg/kg/week, 4 doses in total). No CYT-303-related findings in clinical signs, body weights, food consumption, body temperature, cardiovascular investigations, ophthalmology, clinical chemistry, hematology, coagulation, urinalysis, or anatomic pathology were detected. No treatment-related cytokine release or immunotoxicity was observed in the study. 

Toxicokinetic parameters were assessed by non-compartmental analysis. CYT-303 C_max_ and AUC_0–7d_ exposures generally increased with dose proportionally, and exposures were similar following the first and last doses, indicating no evidence for CYT-303 accumulation in the study. Anti-drug antibody (ADA) screening was conducted to determine whether ADAs were affecting the toxicokinetics of CYT-303. ADA-positive results were reported for only 1/22 CYT-303-treated animals in the low-dose group. This animal did reveal a strong anti-drug response that substantially reduced the CYT-303 exposure. All other treated animals in the study had experienced expected dose-proportional increases. 

In summary, CYT-303 was well tolerated up to the highest dose level (60 mg/kg) tested. The no observed adverse effect level (NOAEL) was determined to be 60 mg/kg under the conditions of this study. 

## 4. Discussion

A CYT-303 NK cell engager was developed by incorporating the GPC3 binder hYP7 humanized antibody and NKp46 binder 09 humanized antibody in the FLEX-NK^TM^ bispecific multifunctional NK engager scaffold. The tetravalent FLEX-NK^TM^ NKE scaffold is comprised of bivalent Fab2 antigen binding domains, and the flexible hinge and linker in CYT-303 enabled sub-nanomolar binding affinities and high avidity binding for both NKp46 and GPC3 targets while simultaneously engaging NK cells and HCC tumors to mediate NK cell-redirected killing of tumors. This NK cell-redirected killing by CYT-303 is further augmented by IgG1 Fc binding and signaling via CD16. This effect, as shown in our studies, is significantly more potent than ADCC mediated by the single anti-GPC3 antibody via Fc binding to CD16 on NK cells against HCC tumors and is an effective way of targeting NK cells against tumors. In addition, the fully functional IgG1 Fc in CYT-303 allows the targeting of additional effector cell types such as macrophages via CD16 and complement fixation to mediate ADCP and CDC against HCC tumors. These multifunctional activities were confirmed by our data. In addition, some tumor cells also express ligands for NKp46, so it would be ideal if the NKp46 binder used in our bispecific engager did not block NKp46 from binding to its natural ligand on tumor cells, which may provide additional tumor ligand signals via NKp46. Our results on the anti-NKp46 mAb 09 binder used in our CYT-303 showed that this binder meets both of these criteria as it does not induce internalization of NKp46 or block the binding of NKp46 to its natural ligands expressed on tumor cells.

In addition, the fully intact Fc provided a reasonable half-life of ~44 h in cynomolgus monkeys. Furthermore, the charged residue mutations at the CH1 and CL interfaces of the anti-NKp46 mAb 1 were introduced to ensure the proper pairing of the light chains to their corresponding heavy chains, and as expected, during process development, no signs of miss-pairing of heavy and light chains were observed since the binding affinity of different batches of CYT-303 to recombinant GPC3 is consistently preserved, and therefore the manufacturability of the product was established (data not shown).

As NKp46 is used to engage CYT-303 on NK cells, it is critical that the binder for NKp46 not induce internalization of NKp46 so that NKp46 would continue to be engaged by CYT-303 on NK cells for subsequent rounds of serial killing of tumors. Our data in NK cell serial killing assays showed the dysfunction of NK cells induced during the serial killing of HCC tumor cells was reversed by CYT-303 in a dose-dependent manner. These results suggest signaling via NKp46 and CD16 plays a role in reversing the dysfunction of NK cells. Although the mechanism of this phenomenon needs thorough investigation, our hypothesis anchors on our preliminary data showing that NKp46 expression is maintained at maximal >85% levels throughout serial killing in dysfunctional NK cells, whereas downmodulation or shedding of activation receptors such as CD16 and NKG2D in these dysfunctional NK cells was detected in the presence of tumors (unpublished data), which could account for the dysfunction of these cells. In addition, it is possible that the tumor ligands for the latter receptor, MICA A/B and ULP, are shed during the serial killing, resulting in poor activation via the NKG2D activation receptor. Therefore, sustained expression of NKp46 in dysfunctional NK cells allows activation by CYT-303 to maintain the serial killing of these cells against HCC. 

GPC3 is a cell-surface glycophosphatidylinositol (GPI)-anchored protein that belongs to the heparan sulfate (HS) proteoglycan family. It can be shed from the tumor cell surface in vivo following cleavage at the Furin cleavage site, which produces the membrane-distal soluble form of GPC3 (sGPC3). This soluble form of GPC3 itself has anti-tumor activity as it can block the activation of Wnt signaling induced by GPC3 and therefore inhibit HCC cell proliferation [23]. The anti-GPC3 antibody hYP7 used in CYT-303 binds to the membrane proximal lobe of the GPC3, which ensures that it can bind to the cell-surface GPC3 expressing tumors even after GPC3 gets cleaved at the N terminal furin cleavage site. Consistent with this observation, it has been shown that hYP7-CAR-T cells have much higher anti-tumor activity in vivo compared to a GPC3 membrane-distal binder (HN3) used in CAR-T cells [24].

Unlike T cell engagers, NK cell engagers have been shown to have a much better safety profile, both preclinically and clinically [25], which is an advantage of NK cell engagers. GPC3 is also a very safe target, as it is a feto-oncogene that is highly expressed in HCC but not in normal adult tissues. Therefore, CYT-303 as an NK engager targeting GPC3 is expected to be very safe. This is confirmed in both the in vitro safety assays and our toxicology studies in cynomolgus monkeys. 

Pharmacokinetic analysis showed dose-proportionate increases in C_max_ and AUC and revealed a half-life of CYT-303 ranging from 39–48 h in cynomolgus monkeys. More importantly, continuous exposure at all dose levels tested can be detected at 168 h post-administration, supporting weekly administration of CYT-303 in the first human clinical trials.

## 5. Conclusions

The potent NK cell-redirected anti-tumor effects observed with CTY-303, together with an outstanding safety profile in vitro in PBMCs and PBNKsand in cynomolgus monkeys as well as a desirable pharmacokinetic profile, warrant clinical investigation of this bispecific, multifunctional NK engager.

## Figures and Tables

**Table 1:** Binding affinity of CYT-303 to target recombinant proteins.

GPC3	NKp46	CD16α
K_D_ = 0.646 nM	K_D_ = 0.183 nM	K_D_ = 642 nM (158 F)
		K_D_ = 147 nM (158 V)

**Table 2:** Pharmacokinetic parameters of CYT-303 in cynomolgus monkeys.

Dose (mg/kg)	C_max_ (μg/mL)	AUC_0–168h_ (μg·h/mL)	T_max_ (h)	T_1/2_ (h)	Vdss (mL/kg)
6	109 ± 16.9	1910 ± 286	0.5	39.0 ± 1.48	136 ± 13.5
20	408 ± 26.9	7740 ± 302	0.5	44.3 ± 0.651	119 ± 8.39
60	1240 ± 175	19,800 ± 1530	0.5	47.6 ± 3.11	115 ± 20.6

## Data Availability

Data are contained within the article.
